# Structural Investigation of DHICA Eumelanin Using Density Functional Theory and Classical Molecular Dynamics Simulations

**DOI:** 10.3390/molecules27238417

**Published:** 2022-12-01

**Authors:** Sepideh Soltani, Shahin Sowlati-Hashjin, Conrard Giresse Tetsassi Feugmo, Mikko Karttunen

**Affiliations:** 1 Department of Physics and Astronomy, The University of Western Ontario, 1151 Richmond Street, London, ON N6A 3K7, Canada; 2 Institute of Biomedical Engineering, University of Toronto, Toronto, ON M5S 3G9, Canada; 3 Department of Chemistry, University of Waterloo, 200 University Ave. West, Waterloo, ON N2L 3G1, Canada; 4 Department of Chemistry, The University of Western Ontario, 1151 Richmond Street, London, ON N6A 5B7, Canada

**Keywords:** eumelanin, 5,6-dihydroxyindole-2-carboxylic acid (DHICA), Molecular Dynamics (MD) simulations, density functional theory (DFT)

## Abstract

Eumelanin is an important pigment, for example, in skin, hair, eyes, and the inner ear. It is a highly heterogeneous polymer with 5,6-dihydroxyindole-2-carboxylic acid (DHICA) and 5,6-dihydroxyindole (DHI) building blocks, of which DHICA is reported as the more abundant in natural eumelanin. The DHICA-eumelanin protomolecule consists of three building blocks, indole-2-carboxylic acid-5,6-quinone (ICAQ), DHICA and pyrrole-2,3,5-tricarboxylic acid (PTCA). Here, we focus on the self-assembly of DHICA-eumelanin using multi-microsecond molecular dynamics (MD) simulations at various concentrations in aqueous solutions. The molecule was first parameterized using density functional theory (DFT) calculations. Three types of systems were studied: (1) uncharged DHICA-eumelanin, (2) charged DHICA-eumelanin corresponding to physiological pH, and (3) a binary mixture of both of the above protomolecules. In the case of uncharged DHICA-eumelanin, spontaneous aggregation occurred and water molecules were present inside the aggregates. In the systems corresponding to physiological pH, all the carboxyl groups are negatively charged and the DHICA-eumelanin model has a net charge of −4. The effect of K${}^{+}$ ions as counterions was investigated. The results show high probability of binding to the deprotonated oxygens of the carboxylate anions in the PTCA moiety. Furthermore, the K${}^{+}$ counterions increased the solubility of DHICA-eumelanin in its charged form. A possible explanation is that the charged protomolecules favor binding to the K${}^{+}$ ions rather than aggregating and binding to other protomolecules. The binary mixtures show aggregation of uncharged DHICA-eumelanins; unlike the charged systems with no aggregation, a few charged DHICA-eumelanins are present on the surface of the uncharged aggregation, binding to the K${}^{+}$ ions.

## 1. Introduction

Eumelanins are black-brown insoluble pigments [1] that are found, for example, in mammalian skin, hair, and eyes [2,3]. Eumelanins have unique properties, such as UV protection [4,5,6,7], antioxidant activity, and free-radical scavenging [8,9]. They have electronic charge carrying properties [10,11], and their amorphous semiconductivity differs from typical polymer semiconductors [12,13].

Eumelanins are highly heterogeneous polymers with no fully established molecular structure [14,15]. Experiments have, however, confirmed that the building blocks of eumelanins are approximately 15–20 Å long, as reported by X-ray measurements [16,17], scanning tunneling microscopy (STM) [18,19], and ultra-high resolution scanning electron microscopy (UHR)-SEM [20]. Furthermore, STM and transmission electron microscopy (TEM) measurements have shown that synthetic crystallized eumelanins can be graphite-like [21]. Their characteristic features have been identified to originate from the aromatic rings with π−π interactions [22], hydrogen bonding, van der Waals interactions, and the high number of carboxylic acid residues with negative charges [23,24].

Even though the exact chemical structure of eumelanin remains elusive, it is well-established that eumelanins are composed of two main building blocks, 5,6-dihydroxyindole-2-carboxylic acid (DHICA) and 5,6-dihydroxyindole (DHI), at various levels of oxidation and linked through covalent C-C bonds into small oligomers [1,12]. The experimentally observed stacking of DHI [25,26,27,28] and bundling of DHICA [3,29] in eumelanin is due to structural disorder; this structural complexity is further emphasized by the fact that the shapes and proportions of monomers differ depending on the source of eumelanin extraction [20,30]. For instance, Pezzella et al. [31] showed that eumelanin obtained from *sepia* melanin is composed of approximately 20% DHI and 75% DHICA, and Magarella et al. [32] demonstrated that the DHICA:DHI ratio for eumelanin obtained from *sepia* melanin depends on the purification procedure.

TEM images of eumelanins with DHICA moiety show aggregation with rod-like granular shapes [3,33,34] with dimers larger than 100 nm. Similarly, SEM analysis of bovine melanosomes [26] shows elongated-shapes. DHI eumelanin shows different behaviour; experiments have reported black-colored onion-like aggregates of 50 nm [3,28] and stacking of planar protomolecules. Featureless UV/Vis absorption, which could be due to non-covalent π−π interaction between the rings in heteroaromatic systems, has been reported in simulations of DHI-eumelanins [28,35,36]. It is plausible that the carboxylate group in DHICA may be responsible for twisting the structure and leading to weak internal interactions and aggregation. In experiments [3], DHICA-eumelanins have shown a distinct band around 310 nm with only little contribution in the visible region of UV/Vis absorption. Based on computer simulations, it has been suggested the that broadening of eumelanin spectra may be due to DHICA monomers with π−π interactions [35]. Compared to DHI, DHICA-eumelanins are lighter in color, have weaker aggregation due to their twisted structure, and have stronger redox and photo-protective properties than DHI-eumelanin [3,37]. It has been reported that DHICA shows potent hydroxyl radical scavenging activity [38] and higher antioxidant activity compared to DHI-eumelanin [9,39].

While several density functional theory (DFT) calculations [28,40,41,42,43,44] and molecular dynamics (MD) simulations [28,35,45] have been reported for DHI-eumelanins, only a few DFT calculations [11,46,47] and one MD simulation [35] of DHICA-eumelanins could be found in the literature review. Although it is not immediately clear why DHICA has received significantly less attention, it can be speculated that it is due to its chemical and structural complexity [48]. DHICA-eumelanin’s complex twisted structure together with the fact that the aggregates it forms are more amorphous and much less well-defined compared to the stacks and onion-like aggregates that DHI-eumelanin forms are most likely the main reasons. In addition, although DHI is less complex, it poses many challenges of its own [28,40,45,49]. From the physical perspective, DHICA is a very interesting molecule that even has potential in bioelectronics [11].

## 2. Materials and Methods

**Figure 1:**
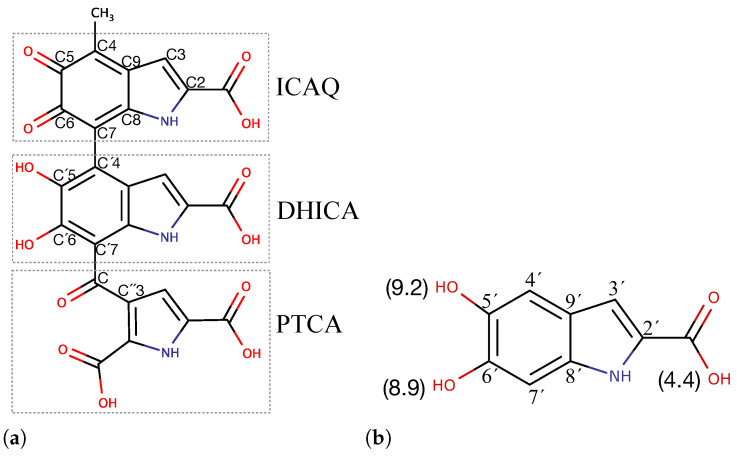
(**a**) The chemical structure of the uncharged eumelanin model (DHICA-eumelanin): the ICAQ (DHICA’s tautomer), 5,6-dihydroxyindole-2-carboxylic acid (DHICA), and pyrrole-2,3,5-tricarboxylic acid (PTCA) moeities. In addition to uncharged DHICA-eumelanin, deprotonated DHICA-eumelanin corresponding to physiological pH was simulated. Due to the PKa value of the DHICA moiety (**b**), all four carboxylic groups deprotonate, and the molecule obtains a net charge of −4 [24,52,53].

The model that is used for the classical atomistic MD simulations in this paper is named DHICA-eumelanin (Figure 1), as it is based on 5,6-dihydroxyindole-2-carboxylic acid (DHICA) moieties. DHICA-eumelanin has three building block units: (1) DHICA’s tautomer, the indole-2-carboxylic acid-5,6-quinone (ICAQ) moiety; (2) the DHICA moiety; and (3) pyrrole-2,3,5-tricarboxylic acid (PTCA), derived from photo-induced oxidative degradation of the DHICA moiety [50,51]. Figure 1a shows the chemical stucture of the model: ICAQ and DHICA are bonded at atoms C7-C4${}^{\prime }$, PTCA is bonded to the DHICA moiety at C-C7${}^{\prime }$, and one of the three carboxylic acids converts to ketone in covalent binding. This model contains the 4-7${}^{\prime }$ DHICA dimer, which is the most stable dimer of DHICA [11]. These units generate a non-planar structure [36]; unlike DHI eumelanin, which has a planar shape [28,40,42,45], they bundle and are not able to fully stack. Hydrogen bonding between the carboxylic groups and π−π interactions between the aromatic rings are the key interactions between the DHICA-eumelanin molecules [14,35].

### 2.1. Geometry Optimization and Validation of the DHICA-Eumelanin Model

First, a model for the DHICA monomer was generated, and its geometry was optimized and validated using DFT calculations. The DFT results are collected in Tables S1–S4, and the atomic coordinates are provided in Table S1. The optimized structure for the DHICA monomer is shown in Figure S1. After geometry optimization (Tables S1–S3), the dipole moment as well as the molecular vibrational modes and their infrared intensities (Table S4) were evaluated; only the vibrational modes of the main functional groups (those larger than 1000 cm${}^{-1}$) are tabulated. Comparison of the bond lengths and angles with the DFT calculations of Powell et al. and Okuda et al. [48,54] in Tables S2 and S3, respectively, show good agreement. The wavenumbers and infrared intensities from Okuda’s results are reported for comparison (Table S5). The vibrational modes are marginally higher than in Okuda’s calculations, which used the Becke three-parameter Lee-Yang-Parr (B3LYP) functional and 6-31G(d,p) basis set and the homogeneous scale factor of 0.975 [54]; it is worth noticing that the DFT calculations and experiments of Okuda et al. [54] showed differences. Potential explanations for this are: (1) the free DHICA monomer in the DFT calculations was in the gas phase, while the experiments were performed using solid samples; (2) the vibrational frequencies were calculated using the harmonic approximation: and (3) the calculated absorption bands were not pure but were a combination of vibrational modes, and as such the intermolecular hydrogen bonds had an influence on the vibrational modes.

The second verification of the model was the calculation of the dipole moment of an individual DHICA monomer. The result, 3.0 Debye, is in good agreement with the DFT calculation of Matta et al., who reported 2.9 Debye [11].

The next step towards classical MD simulations is optimizing the geometry of the DHICA-eumelanin model (ICAQ-DHICA-PTCA) using DFT calculations. The optimized structure is shown in Figure S2. Geometry optimization was carried out at the B3LYP level of theory in the gas phase [55,56,57,58,59,60] combined with the split valence 6-311G(d,p) basis set [61,62,63]. Quantum mechanical calculations were performed using the Gaussian 09 [64] software. The last verification of the optimized geometry was the C6-C7-C4${}^{\prime }$-C5${}^{\prime }$ dihedral angle (see Figure 1a for the structure). The result of 59.2${}^{\circ }$ is in good agreement with Pezzella et al. [36,65], who reported twisted conformations of tautomeric forms of the two-electron oxidation products of DHICA-dimers 4,7${}^{\prime }$-biindolyls (negatively charged carboxylate). They performed geometry optimization at the PBE0/6-31+G(d,p) level of theory and with the dihedral angle varying in the range of 47–63.1${}^{\circ }$ or 112–120${}^{\circ }$. The lowest energy was at the angle of 47.4${}^{\circ }$. On the other hand, our model contains uncharged ICAQ-DHICA-PTCA moieties, and the closest tautomer to all of their structures has the angle of 63.1${}^{\circ }$ (ICAQ-DHICA and the same C5,OH and C6,OH angles). The variation could be due to our system being uncharged, or because in our system the PTCA moiety is bonded to the ICAQ-DHICA moieties, meaning that PTCA could affect the dihedral angle of DHICA-ICAQ.

The LigParGen server [66] was used to obtain the initial parameters of the bonded and non-bonded interactions; the parameterization is compatible with the Optimized Potentials for Liquid Simulations for All Atoms (OPLS-AA) [67,68] force field. The partial charges were computed using electrostatic potential (ESP) [69,70] fitting over the van der Waals surface grid with the Merz-Singh-Kollman (MK) scheme [70,71] at DFT level.

### 2.2. Classical MD Simulations

The MD simulations were performed using the Gromacs 2019.3 package [72] with the OPLS-AA force field [67,68]. This force field has been successfully used for similar systems [45]. The positions of the atoms are shown in Table S6. The bond lengths and angles from the DFT calculations and MD after energy minimization are compared in Tables S7 and S8.

The simple point charge (SPC) model [73] was used for water. An integration time step of 0.5 fs was applied. The Lennard–Jones interactions and the real-space part of threlectrostatic interactions were cut off at 1.0 nm. The particle-mesh Ewald (PME) [74,75] method was used for long-range electrostatics, with the reciprocal-space interactions evaluated on a 0.12 nm grid. The DHICA-eumelanin and water molecules were separately coupled to a heat bath using the V-rescale algorithm [76] at 300 K and 0.1 ps coupling constant. Periodic boundary conditions were used in all directions and hydrogens were constrained using the P-LINCS algorithm [77].

After energy minimization, a pre-equilibration step was performed in the NVT (constant particle number, volume and temperature) ensemble for 2 ns. This was followed by a second pre-equilibration step in the constant particle number, pressure, and temperature (NPT) ensemble using a Parrinello–Rahman barostat [78] at 1 bar with compressibility of $4.5\times 10^{-5}$ bar${}^{-1}$ and time constant of 2.0 ps for 2.0 ns.

To investigate size dependence, four different system sizes and nine different systems were simulated: four (with 2, 4, 27 and 50 DHICA-eumelanins) using uncharged DHICA-eumelanin, four (with 2, 4, 27 and 50 DHICA-eumelanins) with fully deprotonated carboxylic groups, and one mixed system with 25 uncharged and 25 deprotonated DHICA-eumelanins. Potassium counterions were added to maintain overall charge neutrality when necessary.

The uncharged systems were considered as the reference systems. In the range of acidic to physiological pH, all the carboxylic groups of DHICA-eumelanin are ionized [24,52,53] (Figure 1b); system details are provided in Table 1. The sizes of the simulation boxes were chosen to be large enough to ensure that the DHICA-eumelanins were well surrounded by water molecules and the size dependent artificial hydrophobic effect was absent [79,80]. Visualizations were performed using Visual Molecular Dynamics (VMD) [81] and PyMol [82].

## 3. Results and Discussion

**Figure 2:**
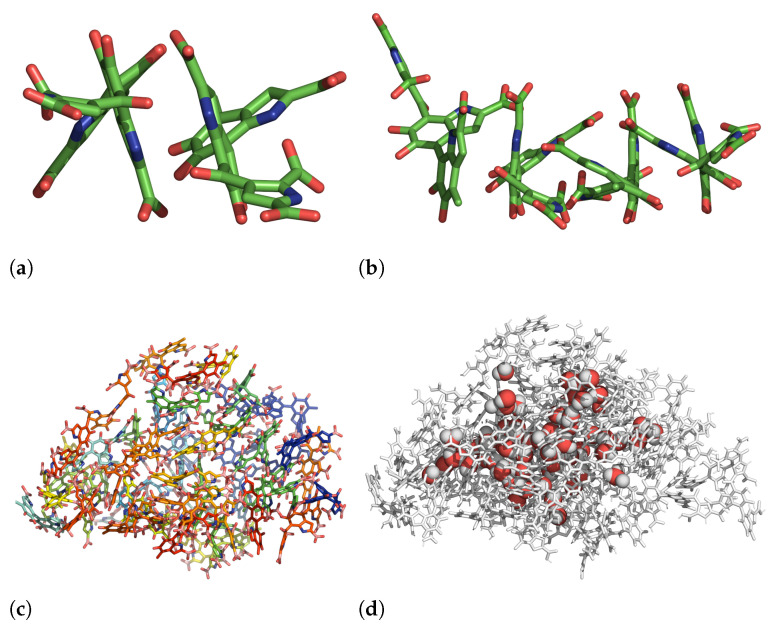
Size dependence of aggregation of uncharged DHICA eumelanin in an aqueous solution. For clarity, water molecules are not shown. (**a**) Two and (**b**) four uncharged DHICA molecules after 1073 ns and 983 ns, respectively. Colours in (**a**,**b**): blue: nitrogen, red: oxygen, white: hydrogen, and green: carbon. (**c**) 50 uncharged eumelanins after 2050 ns, carbon atoms are shown in rainbow to distinguish the residues. (**d**) Same as in (**c**), except the DHICA-eumelanins are shown in gray and water molecules within the aggregate as spheres.

Eumelanins are known to be insoluble in water and to form aggregates. Because DHICA-eumelanin has three twisted moieties/planes, the aggregation process and the resulting structures appear amorphous. When two eumelanins are close enough to attract each other, two of their aromatic rings can stack, and the rest of moieties are free to interact with water molecules and/or neighboring DHICA-eumelanins, Figure 2.

### 3.1. Radial Distribution Functions

**Figure 3:**
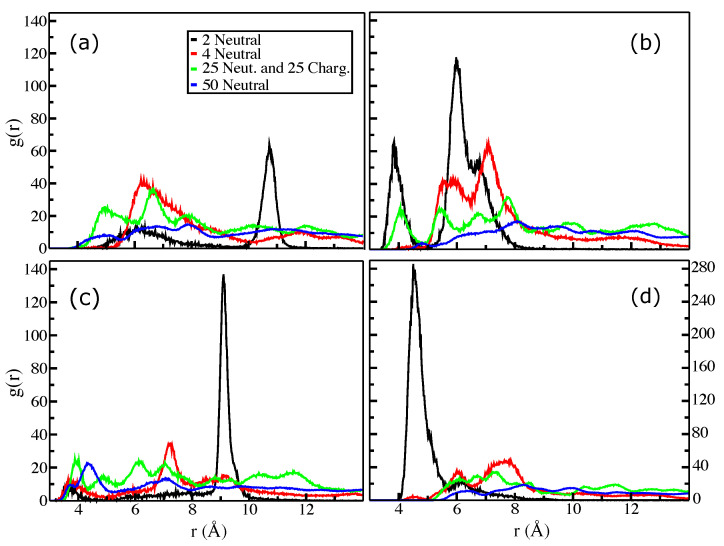
Size dependence of the radial distribution function (RDF). The planes of eumelanin are shown in Figure 1a. RDFs between (**a**) the COMs of the ICAQ moieties, (**b**) the DHICA moieties, and (**c**) the PTCA moieties. (**d**) RDFs between COMs of the DHICA eumelanin molecules. The systems are two uncharged, four uncharged, 25 uncharged and 25 charged, and fifty uncharged eumelanins; see details in Table 1.

**Figure 4:**
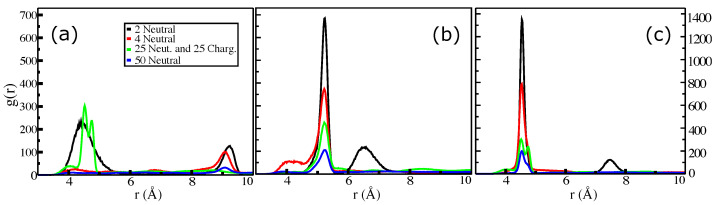
Size dependence of the radial distribution function (RDF). The planes of eumelanin are shown in Figure 1a. RDFs between the COMs of (**a**) the ICAQ and PTCA moieties, (**b**) the DHICA and PTCA moieties, and (**c**) the ICAQ and DHICA moieties. The systems are two uncharged, four uncharged, 25 uncharged and 25 charged, and fifty uncharged eumelanins; see details in Table 1.

Figure 3 and Figure 4 show the radial distribution functions (RDF) between the centers of masses (COMs) of selected planes (see Figure 1a for the structures) from the last 100 ns. RDFs for systems of two, four, and fifty uncharged DHICA-eumelanins were computed. The RDFs for systems of two DHICA-eumelanins are shown in black. As Figure 3a shows, in the system of two DHICA-eumelanins the ICAQ moieties are at distances of 6 and 10.6 Å. The DHICA moieties of two DHICA-eumelanins, however, are in the range of π−π interactions, with the first peak at 3.8 Å followed by peaks at further distances of 6 Å and 6.8 Å; see Figure 3b. The PTCA moieties have the closest peak in the range of π−π interactions, at 3.6 Å, Figure 3c. Classical MD simulations do not contain electrons, and hence π−π interactions have only an operational definition. It is typically based on the intermolecular distance and sometimes supplemented by an angle defined between the centroids (here, COMs) of the two rings [83]. The typical distance criterion is 3.2–4.0 Å and the centroid–centroid distance is then slightly longer than the intermolecular distance, as the rings are slightly shifted [83] (parallel-displaced configuration [84]); see the ring orientations in Figure 2.

The RDFs for the systems of four DHICA-eumelanins are shown in red in Figure 3. Because these systems are larger and have more structural flexibility than the systems with two DHICA-eumelanins, the RDFs show more structure. The first and dominant distances are generally further than in the systems of two DHICA-eumelanins, and only the PTCA moieties, shown in Figure 3c, have their first peak in the range of π−π interactions at 3.7 Å. The dominant peak, however, is at 7.2 Å. The shift to larger distances continues with the largest system of 50 neutral DHICA-eumelanins; similarly to the case of four DHICA-eumelanins, only the case of PTCA shows a peak in the range of π−π interactions at 3.7 Å, with the dominant peak at 4.3 Å.

We investigated the RDFs between the COMs of the ICAQ and the PTCA moieties (Figure 4a), the DHICA and the PTCA moieties (Figure 4b), and the ICAQ and the DHICA moieties (Figure 4c). All the systems show multiple peaks with the primary peak being mostly in the range of about 4.5–5.2 Å. In all cases, there is a minor structure around 4 Å which is in the range of π−π interactions.

In summary, the RDFs revealed the possibility of π−π interactions. The other mechanism that promotes aggregation is hydrogen bonding between the carboxylic acids of two neighboring uncharged DHICA-eumelanins. The DHICA-eumelanin structure is not rigid. Apart from being able to rotate around each of the bonds linking the planes, each planar moiety may bend slightly. Due to the twisted structure, at most only two aromatic rings are able to interact via π−π interactions, while the remaining planes cannot form π−π interactions with the same neighboring eumelanin; these may interact via H-bonds or van der Waals interactions with other DHICA-eumelanins or water molecules. These situations are shown in Figure 2a,b with two and four eumelanins.

### 3.2. Aggregation

**Figure 5:**
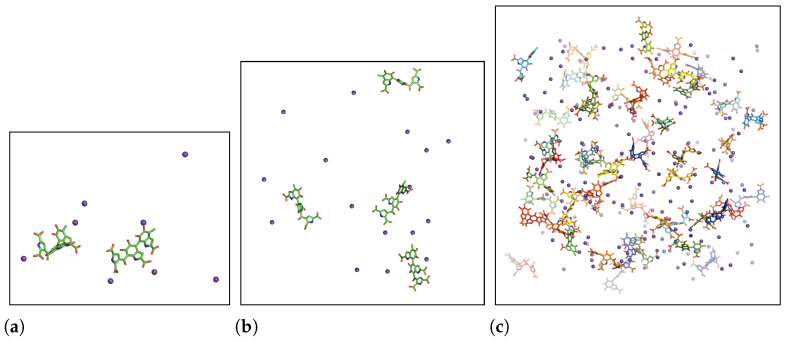
Aggregation of charged DHICA eumelanin molecules. Water molecules are not shown for clarity. Colours in (**a**,**b**): blue: nitrogen, red: oxygen, white: hydrogen, green: carbon. The potassium ions are shown in purple. (**a**) Two charged DHICA eumelanins after 1000 ns, (**b**) four charged DHICA eumelanins after 1100 ns, and (**c**) 50 charged DHICA eumelanins after 1361 ns. In (**c**). rainbow colouring is used to distinguish the residues.

Aggregation of eumelanins in aqueous solutions was studied under conditions corresponding to pH = 7.4 using systems of two, four, and 50 charged DHICA-eumelanins. The details of the respective systems are provided in Table 1. The charged (protonated) eumelanin has four carboxylic acid groups (Figure 1a), which have been indicated as potential binding sites for drugs [85,86]. No aggregation occurred in the charged systems, as shown in Figure 5. At higher concentrations, however, pairing of eumelanins via π−π interactions was observed, as shown in Figure 5c.

**Figure 6:**
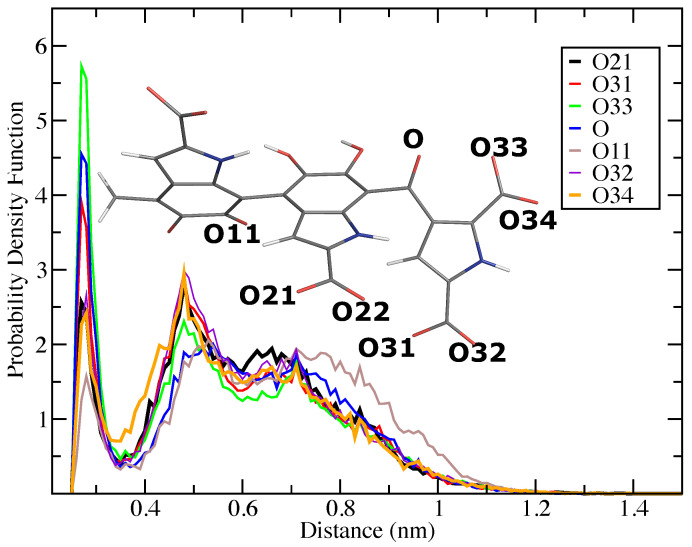
Distribution of K${}^{+}$- charged DHICA’s oxygen distances for the system of four charged DHICA-eumelanins. O21, O31, and O33 stand for the oxygen atoms of the hydroxyl group in carboxylic anions, and O22, O32, and O34 are the oxygen atoms of the carboxyl group in the carboxylic ions group. O is the oxygen atom of the ketone in PTCA, and O11 was chosen as reference.

Next, the distances between the counterions (K${}^{+}$) and the oxygen atoms in the system of four charged DHICA-eumelanins were examined in the time window of 1000–1100 ns. The selected distance distributions (the probability density functions) are shown in Figure 6. The results show that the K${}^{+}$ ions favour interactions with the oxygen atoms of PTCA. In addition, the first shell between the K${}^{+}$ ions and the oxygen atoms of the PTCA moieties is at 2.7–2.8 Å, which is in the same range (2.73–2.79 Å) as the first shell of the water oxygens and the K${}^{+}$ ions reported in experiments [87]. Thus, K${}^{+}$ ions interact with water molecules as well as with DHICA-eumelanins.

**Figure 7:**
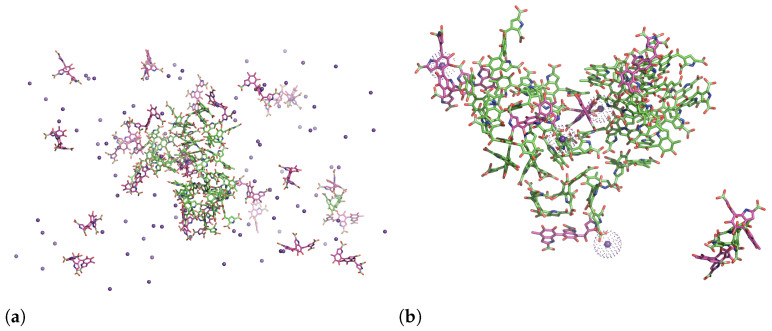
Mixture of 25 uncharged (green) and 25 charged (pink) DHICA eumelanins. K${}^{+}$ ions (purple spheres) were added to the system to neutralize it. (**a**) Snapshot of the system at 1971 ns and (**b**) a zoomed-in view showing charged DHICA eumelanins within 3 Å of uncharged ones. Water molecules are not shown for better clarity.

Snapshots from a mixture of 25 uncharged and 25 charged DHICA eumelanins after about 1900 ns are shown in Figure 7. As the figure shows, the uncharged eumelanins (green) become shielded by the charged ones (pink). As Figure 7a shows, a number of the charged molecules and counterions remain dispersed in the solution. Figure 7b shows a zoomed-in view displaying aggregated uncharged eumelanins and charged eumelanins that are within 3 Å of uncharged eumelanins as well as the K${}^{+}$ ions that are within 3 Å of charged eumelanins. The K${}^{+}$ ions that bind with carboxylic acids are shown as dots in order to identify them better. During the aggregation process, one of the charged eumelanins became trapped inside the cluster. The figure shows that due to hydrophobic sites in the uncharged molecules, they prefer to interact with themselves rather than with water molecules. The charged eumelanins, however, tend to form hydrogen bonds with water molecules or bind with the K${}^{+}$ ions. In addition to the above, a few of the charged eumelanins are in contact with uncharged eumelanins via π−π interactions.

The RDFs for the mixed systems can now be re-examined with the above behaviour in mind. The green lines in Figure 3 and Figure 4 show the mixed systems. Comparison of the RDFs from the system of 50 neutral DHICA-eumelanins (blue lines in Figure 3 and Figure 4) and the snapshots in Figure 7 makes it clear that the uncharged DHICA-eumelanins are compressed by the charged DHICA-eumelanins in the mixed systems.

### 3.3. Dihedral Angles

The DHICA-eumelanin protomolecule has a twisted structure, as is clear from Figure 4. Previous quantum computations [11,36,65] have shown that the dihedral angle between the DHICA (5,6-dihydroxyindole-2-carboxylic acid) and ICAQ moieties (see Figure 1a for the definitions of the groups) is in the range of 47–63.1${}^{\circ }$. Our DFT calculations in the gas phase resulted in 60.3${}^{\circ }$, which is in good agreement with Pezzella’s results [36], though higher than Matta’s [11] DFT result of $50^{\circ }$ for the DHICA dimer (see Section 2.1 *Geometry optimization and validation* for more details).

**Figure 8:**
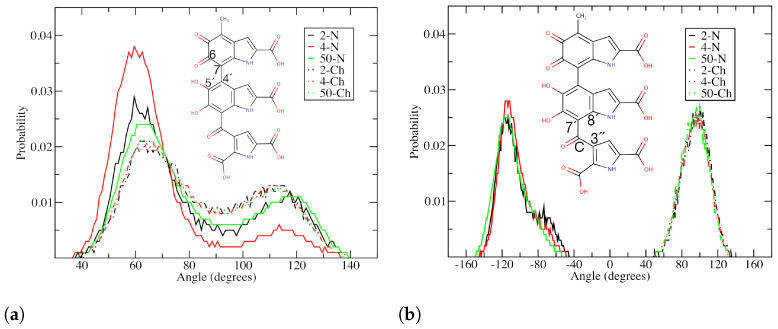
Dihedral angle distributions for all the systems over the last 100 ns: (**a**) C6-C7-C4${}^{\prime }$-C5${}^{\prime }$ atoms and (**b**) C8${}^{\prime }$-C7${}^{\prime }$-C-C3${}^{\prime \prime }$ atoms. Solid lines: uncharged molecules. Dotted lines: charged molecules.

The dihedral angle distributions from the classical MD simulations in explicit water are shown in Figure 8. The dihedral angle between the DHICA group and its tautomer, ICAQ, is defined by the C6-C7-C4${}^{\prime }$-C5${}^{\prime }$ atoms (Figure 1a). The results are shown in Figure 8a. In the uncharged systems (solid lines) of the two protomolecules the most probable angle is around 60${}^{\circ }$, with a second peak present around 120${}^{\circ }$. The amplitude of the first peak decreases as the number of molecules increases. The second peak displays opposite behaviour. The main peak is around $60^{\circ }$, while the second peak is shifted slightly to around 110${}^{\circ }$. The result of $60^{\circ }$ is in very good agreement with the experiments of Corani et al. [88].

**Figure 9:**
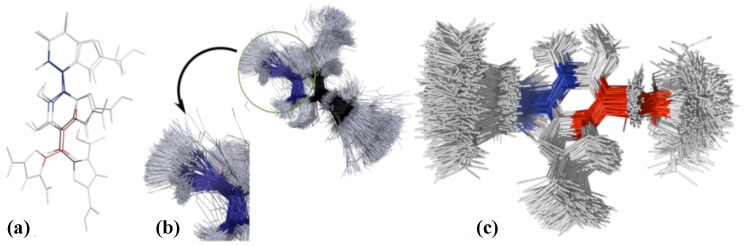
(**a**) Snapshots of two single-aligned charged and uncharged DHICA-eumelanins: (**b**,**c**) 100 superimposed structures over the last 100 ns from the systems of four uncharged (**b**) and four charged (**c**) DHICA-eumelanins, respectively. The angle C6-C7-C4${}^{\prime }$-C5${}^{\prime }$ is shown in blue, C8${}^{\prime }$-C7${}^{\prime }$-C-C3${}^{\prime \prime }$ for the charged DHICA-eumelanin is in red, and for uncharged is in black.

Considering that there are three twisted planes, the next dihedral angle to investigate is the one between the DHICA and PTCA moieties (Figure 1a). The results are shown in Figure 8b. The geometries of individual uncharged and charged DHICA-eumelanins are presented as aligned structures in Figure 9a. The dihedral angles for the uncharged systems vary between $-50^{\circ }$ to $-145^{\circ }$, with a peak at $-110^{\circ }$. For the charged systems, the dihedral angles are in the range of 50–130${}^{\circ }$, with a peak at around 100${}^{\circ }$.

To better understand the results in Figure 8, Figure 9a shows the ICAQ-DHICA (C6-C7-C4${}^{\prime }$-C5${}^{\prime }$ atoms) dihedral angle in blue. For the DHICA-PTCA angle (C8${}^{\prime }$-C7${}^{\prime }$-C-C3${}^{\prime \prime }$ atoms), black is used for the uncharged and red for the charged DHICA-eumelanin. Snapshots of 100 superimposed structures from the system with four uncharged DHICA-eumelanins are shown in Figure 9b, and Figure 9c shows the same for the corresponding charged system. The main difference in their behaviour is that the charged DHICA-eumelanins are soluble in water and that the PTCA moiety is in favor of interacting with the K${}^{+}$ ions. This is likely the the reason for the DHICA-PTCA dihedral angle distribution being narrower in the charged systems.

### 3.4. Hydrogen Bonding

**Figure 10:**
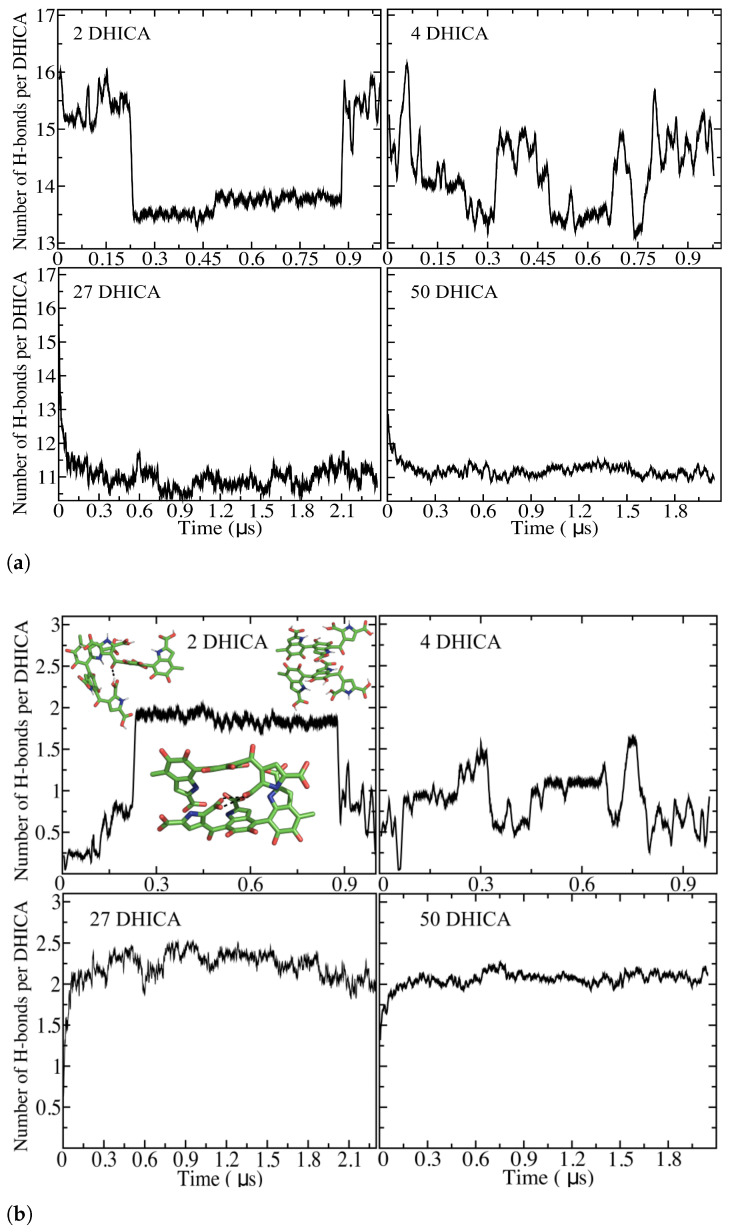
Time evolution of the number of hydrogen bonds per monomer for two, four, 27, and fifty DHICA-eumelanin systems (**a**) between DHICA eumelanin and water and (**b**) between the eumelanin molecules.

One of the most key molecular interactions is hydrogen bonding. We computed H-bonds based on a donor–acceptor distance of <3.5 Å and hydrogen donor–acceptor angle of $<\;25^{\circ }$. Figure 10a shows the average number of H-bonds between the DHICA-eumelanins and water per DHICA-eumelanin molecule. In the small systems of two and four molecules (the upper panels), the number fluctuates, while in the two larger systems (lower panels) it settles to about 11.5. The number of H-bonds between the DHICA-eumelanins settles to approximately two after 60 ns (Figure 10b).

The H-bonding properties are a consequence of the complex twisted structure, as is apparent from the analysis of the dihedral angles and the orientations of the different moieties with respect to each other. Certain potentially favourable interactions are not possible due to the steric constraints and the orientations of the different groups. The complexity of the situation is clear from Figure 2. Figure 2a,b shows the non-planar structures; as Figure 2d shows, water is trapped within the complex.

We analyzed all the H-bonds in the system; one special feature is that the ICAQ group forms on average only about 0.07 intermolecular H-bonds with the neighboring molecules’ ICAQ groups, while all the other intermolecular combinations have either around 0.3 (PTCA-PTCA, DHICA-DHICA and PTCA-ICAQ) or even higher (>0.5) H-bonds per moiety (ICAQ-DHICA and PTCA-DHICA). That is, the DHICA is the key part for intermolecular H-bonding. Regarding the about two H-bonds/molecule (intermolecular), that number is enough to provide the aggregates with enough stability against thermal fluctuations to allow them to form.

As for intramolecular bonding, the dominant bond is between PTCA and ICAQ, although its contribution is very small; over the simulation time, the number of bonds fluctuates between 0.02 and 0.06.

The presence of water is critical in these systems. For example, when compared to the DFT calculations of Matta et al. [11], which were carried out in the absence of water, intramolecular H-bond networks form in either a zig-zag or helical fashion. In addition, their systems consisted of DHICA moieties only. The case here is very different; due to the presence of water, no such networks appear.

### 3.5. Dipole Moment

The dipole moment for the uncharged DHICA-eumelanin model was calculated in the gas phase with the B3LYP/6-311G(d) basis sets and ESP fitting [69,70] following the Merz-Singh-Kollman (MK) scheme [70,71] and a 500 self-consistent field cycles. The calculation yielded 11.0 Debye. For comparison, the same system was optimized with the ωB97XD/6-311G(d,p) basis sets without ESP fitting and 500 self-consistent field cycles. The result was 11.5 Debye. Both methods provided higher values than the results for dimeric DHICA reported by Matta et al. [11], who obtained 5.8 Debye. However, they obtained 12.7 Debye for tetrameric DHICA (four carboxylic acids). Our result for the trimeric form is between these two values. In addition, differences originate from the carbonyl groups in ICAQ and from the PTCA moiety due to polarity of the carboxylic acids.

## 4. Conclusions

We studied the aggregation of DHICA-eumelanin using a combination of multi-microsecond MD simulations and DFT calculations. The latter were performed to parameterize and verify the model and to determine the dipole moments. Classical MD simulations were performed at different concentrations of uncharged DHICA-eumelanins, charged DHICA-eumelanins (the protonation state corresponding to about pH 7.4), and a mixture of the two. The DFT calculation of the dihedral angle of uncharged ICAQ-DHICA moieties in the gas phase resulted in approximately $60^{\circ }$, which is in good agreement with the prior DFT studies of Pezzella et al., who used negatively charged ICAQ-DHICA moieties [36]. The dipole moment of uncharged DHICA-eumelanin in the gas phase was carried out at B3LYP/6-311G(d) and ωB97XD/6-311G(d,p) levels. The calculations resulted in 11–11.5 Debye. This value is above the DHICA-dimer dipole moment of 5.8 Debye and below the tetramer-DHICA value of 12.7 Debye reported by Matta et al. [11] using DFT calculations at ωB97XD-D/6-31G+ level of theory.

In the classical MD simulation with explicit water, the uncharged DHICA-eumelanins were aggregated to bundles. Due to their twisted structure, only minor or no π−π interactions were present. Instead, hydrogen bonds formed. Because the interactions between the molecules were relatively weak, water was trapped inside the aggregates at higher concentrations. This is very different from the aggregation of DHI-eumelanins, which form well-defined tight stacks with no water inside the aggregates [28,45].

The simulation results show that, upon aggregation, the average number of H-bonds between the individual DHICA-eumelanins settles to about two H-bonds/molecule. The number of H-bonds between the DHICA-eumelanins and water plateaus at around 11/DHICA-eumelanin. The dihedral angle distribution between the DHICA and ICAQ moieties in water solution is semi-bimodal, with a peak around $60^{\circ }$. This is in very good agreement with the experiments of Corani et al. [88] (≈$60^{\circ }$), who studied 4-7${}^{\prime }$ DHICA moiety dimers in water, though higher than Matta’s [11] results for oligomers of DHICA moieties $50^{\circ }$ using ωB97X-D/cc-pVTZ level of theory.

All carboxylic acids in DHICA-protomolecules become ionized at physiological pH, and the protomolecule attains a net charge of −4. The charged protomolecules favour interactions with water and potassium ions rather than with themselves. Distributions of K${}^{+}$ ions around the DHICA-eumelanins show the first shell at ≈2.7 Å, which is in the same range as the experimentally measured potassium–water (using water’s oxygen) first shell at 2.73–2.79 Å [87]. The reason that PTCA moieties contribute to ion binding more than DHICA or ICAQ moieties could arise from the fact that the dihedral angle distribution between PTCA and DHICA moieties for charged DHICA-eumelanins is around $110^{\circ }$. However, the DHICA and ICAQ moieties are closer together, and the dihedral angles are $\approx 65^{\circ }$ to minimize electrostatic interactions, which is slightly higher than in the case of uncharged DHICA-eumelanin.

The systems of mixed charged and uncharged DHICA-eumelanins show aggregation of uncharged eumelanin, with charged DHICA-eumelanins H-bonding to them. Potassium ions bind to the carboxylic acids of the charged molecules. The results of this study show that DHICA-eumelanins have structural disorder, as well as that charged DHICA-eumelanins, unlike the uncharged ones, are soluble in an aqueous solution with potassium ions. Potassium ions prevent fast aggregation. This could potentially allow for investigations of absorption profiles of natural eumelanin without scattering effects. In addition, the experimentally observed aggregates in naturally occurring eumelanin are most likely composed of uncharged DHICA, with charged molecules H-bonding to them at the surfaces of the aggregates. Finally, the bundled and disorderd shapes of the aggregates suggest that structural disorder should be considered in models for drug–eumelanin binding.

## Figures and Tables

**Table 1:** Details of the simulated systems.

Number of Eumelanins (nm${}^{3}$)	Number of Water Molecules	Box Size (nm${}^{3}$)	Counterions (K${}^{+}$)	Duration (μs)
2 uncharged	4218	${\left(5.07\right)}^{3}$	0	1.073
4 uncharged	5640	${\left(5.59\right)}^{3}$	0	0.983
27 uncharged	6341	7.16×7.30×7.14	0	2.237
50 uncharged	20,000	${\left(8.60\right)}^{3}$	0	2.050
2 charges	3487	${\left(4.75\right)}^{3}$	8	1.000
4 charges	4501	${\left(5.18\right)}^{3}$	16	1.100
27 charges	6272	${\left(5.87\right)}^{3}$	108	3.802
50 charges	19,800	${\left(8.57\right)}^{3}$	200	1.361
25 uncharged-25 charged	19,900	11.74×7.39×7.32	100	1.974

## Data Availability

Parameters for the DHICA-eumelanin protomolecule are available at https://github.com/SoftSimu/melanin (accessed on 15 September 2022) including Gromacs compatible gro and itp files.

## Supplementary Materials

The following supporting information can be downloaded at https://www.mdpi.com/article/10.3390/molecules27238417/s1. Figure S1: DHICA monomer structure. Figure S2: Geometry optimized DHICA-eumelanin chemical structure (ICAQ-DHICA-PTCA). Table S1: Atom types and coordinates of monomer DHICA’s Dreyer structure (in  Å). Table S2: Bond lengths of monomer DHICA’s Dreyer structure (in  Å) compared with Okuda’s [54] and Powell’s [48] results. Table S3: Comparison of the angles of the DHICA monomer with Okuda’s [54] and Powell’s [48] results. Table S4: Calculated molecular vibrational intensity for DHICA monomer. Table S5: Calculated molecular vibrational intensity for DHICA monomer of Okuda’s results for comparison with Table S4. Table S6: Atom types and coordinates of optimized DHICA-eumelanin (in  Å). Table S7: Comparison of bond lengths of DHICA-eumelanin calculated using DFT and MD simulations with explicit water model. Table S8: Comparison of angle degrees of DHICA-eumelanin calculated using DFT and MD simulations with explicit water model.

## Abbreviations

The following abbreviations are used in this manuscript:

DHICA	5,6-DiHydroxyIndole-2-Carboxylic Acid
DHI	5,6-DiHydroxyIndole
PTCA	Pyrrole-2,3,5-TriCarboxylic Acid
DFT	Density Functional Theory
STM	scanning tunneling microscopy
UHR-SEM	ultra-high resolution scanning electron microscopy
MD	Molecular Dynamics
OPLS-AA	Optimized Potentials for Liquid Simulations for All Atoms
SPC	Simple Point Charge
PME	Particle-Mesh Ewald
P-LINCS	Parallel LINear Constraint Solver
NVT	constant particle Number, Volume, and Temperature
NPT	constant particle Number, Pressure, and Temperature
VMD	Visual Molecular Dynamics
RDF	Radial Distribution Functions
COM	Center of Mass
ESP	ElectroStatic Potential
B3LYP	Becke three-parameter Lee–Yang–Parr
MK	Merz–Singh–Kollman
