# Supplementary material for: Structural Investigation of DHICA Eumelanin Using Density Functional Theory and Classical Molecular Dynamics Simulations
Source: Molecules. 2022 Dec 1;27(23):8417. doi: 10.3390/molecules27238417 (PMC9738096; doi:10.3390/molecules27238417)
Supplement: Supplementary file 1 [file molecules-27-08417-s001.zip › molecules-2027833-supplementary.pdf]

SUPPORTING INFORMATION

Structural investigation of DHICA eumelanin using density functional theory and classical molecular dynamics simulations

Sepideh Soltani,† Shahin Sowlati-Hashjin,‡ Conrard Giresse Tetsassi Feugmo,¶ and Mikko Karttunen*,†,§

†*Department of Physics and Astronomy, The University of Western Ontario, 1151 Richmond Street, London, Ontario N6A 3K7, Canada*

‡*Institute of Biomedical Engineering, University of Toronto, Toronto, Ontario M5S 3G9, Canada*

¶*Department of Chemistry, University of Waterloo, 200 University Ave. West, Waterloo, Ontario N2 3G1, Canada*

§*Department of Chemistry, The University of Western Ontario, 1151 Richmond Street, London, Ontario N6A 5B7, Canada*

E-mail: mkarttu@uwo.ca

Molecular structure of the DHICA monomer was geometry-optimized by DFT calculations using Gaussian 09 (Ref. [64] in the main manuscript). The atom types and coordinates are shown in Table S1. The bond lengths and angles evaluated by Okuda (Ref. [54] in the main manuscript) and Powell (Ref. [48] in the main manuscript) are provided in Tables S2 and S3, respectively, for reference. As the tables show, the results are in good agreement.

The average difference between the DFT calculations and MD simulations after energy minimization was 1.42% for the bond lengths and 1.37% for the angles.

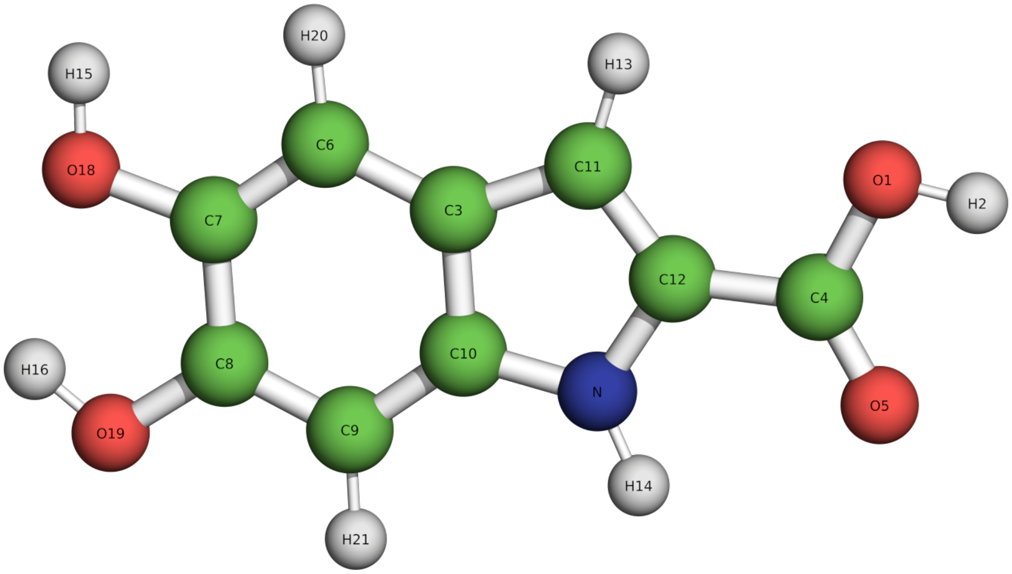

Figure S1: DHICA monomeric structure

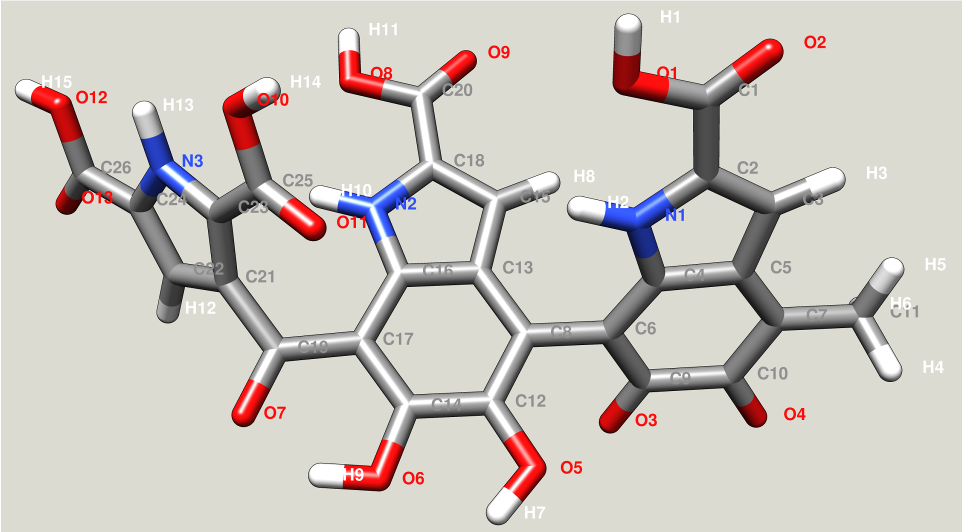

Figure S2: Eumelanin chemical structures which serve as DHICA-eumelanin model structure in this article

Table S1: Atom types and coordinates (unit: Å) of DHICA monomer optimized using DFT calculation.

	**Atom Name**	**X(Å)**	**Y (Å)**	**Z(Å)**
1	O	3.95050	1.22780	0.18430
2	H	4.90800	1.02960	0.21480
3	C	-0.24050	0.71090	0.05100
4	C	3.37930	0.00790	0.15880
5	O	4.05530	-1.00850	0.17490
6	C	-1.49980	1.34660	0.01700
7	C	-2.64300	0.54800	-0.01660
8	C	-2.55020	-0.84070	-0.01740
9	C	-1.32280	-1.49360	0.01470
10	C	-0.18110	-0.68370	0.04900
11	C	1.10070	1.18210	0.09130
12	C	1.94930	0.08900	0.11420
13	H	1.41910	2.21710	0.10390
14	H	1.50030	-1.97960	0.09340
15	H	-3.80190	2.06470	-0.04390
16	H	-4.41520	-0.93560	-0.06750
17	N	1.14900	-1.03070	0.08590
18	O	-3.89810	1.09680	-0.04940
19	O	-3.69200	-1.59380	-0.05010
20	H	-1.55840	2.42880	0.01820
21	H	-1.26470	-2.57600	0.01370

Table S2: Comparison of bond lengths (Å) calculated using DFT for the DHICA monomer in this paper and in the papers of Okuda (Ref. [54] in the main manuscript) and Powell (Ref. [48] in the main manuscript).

Bond	Present	Okuda	Powell
O1-C4	1.354040	1.356	1.368
O5-C4	1.214197	1.221	1.225
C6-C3	1.411795	1.414	1.414
C3-C10	1.424280	1.403	1.403
C3-C11	1.423073	1.425	1.421
C4-C12	1.454581	1.456	1.453
C6-C7	1.374746	1.378	1.380
C7-C8	1.424782	1.427	1.428
C8-C9	1.380375	1.384	1.387
C9-C10	1.401056	1.403	1.403
C10-N17	1.368609	1.370	1.372
C7-O18	1.380508	1.380	1.384
C8-O19	1.359174	1.361	1.365

Table S3: Comparison of bond angles (deg.) calculated using DFT for the DHICA monomer in this paper with the results of Okuda (Ref. [54] in the main manuscript)) and Powell (Ref. [48] in the main manuscript).

Bond	Present	Okuda	Powell
C6-C3-C10	118.9	118.9	119.3
C6-C3-C11	134.0	134.0	133.5
C10-C3-C11	107.1	107.1	107.2
O1-C4-C5	122.7	122.8	112.8
O1-C4-C12	113.1	113.3	112.9
O5-C4-C12	124.2	123.9	124.2
C3-C6-C7	118.6	118.6	118.3
C6-C7-C8	121.7	121.8	121.7
C6-C7-O18	124.9	124.8	124.8
C8-C7-O18	113.4	113.4	113.6
C7-C8-C9	120.8	120.9	121.2
C7-C8-O19	119.2	119.1	118.9
C9-C8-O19	120.0	120.0	119.8
C8-C9-C10	117.6	117.4	117.2
C3-C10-C9	122.3	122.4	122.3
C3-C10-N17	107.4	107.4	107.3
C9-C10-N17	130.3	130.2	130.5
C3-C11-C12	107.0	107.0	107.0
C4-C12-C11	132.3	132.6	132.0
C4-C12-N17	118.5	118.2	118.9
C11-C12-N17	109.1	109.2	109.1
C10-N17-C12	109.3	109.3	109.4

Table S4: Calculated vibrational wave numbers and IR intensities for the DHICA monomer in the current article. Only vibrational modes of the main functional groups are tabulated (larger than 1000 cm^{-1}). For the labels of the atoms, see Fig. 1. The following abbreviations are used: ν, stretching; δ, in-plane bending; Skel., Skeleton; def., deformation.

Wavenumber present (cm^{-1})	IR Intensity (km mol^{-1})	Vibrational modes
3852.9	80.9	νO18H15
3779.5	146.2	νO19H16
3773.3	135.0	νO1H2
3653.7	103.1	νN17H14
3253.6	0.5	νC11H13
3196.5	2.9	νC9H21
3159.1	14.6	νC6H20
1777.5	569.6	νO5C4, δO1H2
1686.3	22.8	Benzene breathing, δC9H21, δC6H20, δO19H16
1629.6	11.3	Benzene breathing, δO18H15, Pyrrole def., δN17H14, δO19H16
1574.8	267.8	Skel. def., δO19H16, δO1H2
1556.2	63.9	δO18H15, δO19H16, Skel. def., δN17H14
1507.9	15.1	Benzene breath., Pyrrole breath. δC9H21, δN17H14, δC6H20, δO19H16
1451.3	112.8	Pyrrole def., νN17C12, δO19H16, δC9H21, δN17H14
1429.0	2.9	δN17C12, δO19H16, δO1H2
1390.8	14.2	Benzene breathing, νN17C10, δN17C12, δC9H21, δC6H20, δO19H16
1375.0	17.6	δO18H15, δO19H16, δC9H21, δC6H20
1342.1	360.5	Pyrrole breathing, δO1H2, δO1H2, δO19H16,
1266.8	235.0	δN17C12, δO19H16, δC11H13,δO1H2
1249.3	67.6	Skel. def., δO19H16, δC9H21, δC6H20
1232.0	82.7	Skel. def., δO19H16,δC9H21, δO1H2, δO18H15,δC6H20
1200.6	185.4	δO19H16, δC11H13, δC9H21 δO1H2, δO18H15, δC6H20
1180.6	25.1	Benzene breathing, δO1H2 δC9H21, δC6H20, δO18H15
1157.9	530.2	Benzene breathing, δN17H14, δC11H13 δO1H2, δO18H15, δC6H20
1142.9	213.4	Skel. def., δN17H14, δO18H15
1109.3	24.3	Pyrrole def., δN17H14, δO18H15

Table S5: Calculated molecular vibrational intensities for the DHICA monomer from Okuda's article (Ref. [54] in the main manuscript)).

Wavenumber (cm^{-1})	IR Intensity (km mol^{-1})	Vibrational modes
3750.5	68.0	νO18H15
3680.7	129.2	νO19H16
3674.8	116.7	νO1H2
3579.9	101.3	νN17H14
3192.1	0.7	νC11H13
3138.0	3.0	νC9H21
3097.2	15.0	νC6H20
1749.9	517.3	νO5C4
1657.4	23.4	Benzene def.
1600.3	9.5	Skel. def.
1549.4	278.9	Pyrrole breathing, Benzene def.
1529.1	68.2	Skel. def., νC8O19
1485.7	11.1	Skel. def., νC7O18
1428.3	124.4	νN17C12
1406.9	2.7	Skel. def., νC4O1, δO19H16
1371.3	15.4	Pyrrole def., Benzene breathing, νC8O19 , νC7O18
1349.3	9.3	δO18H15, δO19H16
1320.4	435.8	Skel. breathing, δO1H2, νC8O19, νC4O1
1243.4	195.8	δN17H14, δC11H13, νC8O19
1226.0	92.7	δC6H20
1209.8	54.5	δO19H16, δO1H2, νC8O19, Skel. def.
1176.3	197.7	δC9H21, δO18H15, δO19H16, δO1H2
1157.8	18.7	δN17H14, δC9H21
1137.3	423.8	δO1H2, δC11H13, νC4O1
1121.5	273.5	δO18H15, δN17H14, νC7O18
1092.7	19.8	δC11H13, δN17H14, νC4O1

Table S6: Atom types and coordinates (unit:Å) of DHICA-eumelanin (dopachrome-DHICA-PTCA). The molecular structure is optimized using DFT calculations.

Atom Name	X	Y	Z
H	-2.21913	4.42918	-2.31489
O	-2.34932	3.56601	-1.90581
C	-3.64513	3.48385	-1.55653
C	-3.94859	2.19151	-0.91221
O	-4.44611	4.35538	-1.74681
N	-2.98028	1.21241	-0.72646
C	-5.14571	1.79446	-0.43531
H	-2.01001	1.30505	-0.97801
C	-3.53568	0.13001	-0.09518
H	-6.05122	2.3769	-0.46797
C	-4.96281	0.46139	0.11203
C	-2.93141	-1.02804	0.27028
C	-5.82843	-0.39554	0.69383
C	-1.50045	-1.28096	0.03051
C	-3.76273	-2.02059	0.95424
C	-5.28818	-1.70571	1.1342
C	-7.28113	-0.13801	0.91714
C	-1.09232	-2.34522	-0.73784
C	-0.50369	-0.42965	0.57667
O	-3.34314	-3.05519	1.40972
O	-5.99622	-2.541	1.63849
H	-7.88117	-0.88685	0.39324
H	-7.57698	0.85391	0.57579

Continued on next page

Table S6 – continued from previous page

Atom Name	X	Y	Z
H	-7.52313	-0.23578	1.97863
O	-1.99556	-3.16263	-1.31565
C	0.28931	-2.58741	-0.97244
C	-0.59029	0.69928	1.43887
C	0.86096	-0.66024	0.3225
H	-1.51689	-3.8843	-1.7356
O	0.53716	-3.66111	-1.7256
C	1.29735	-1.74708	-0.4822
H	-1.47837	1.15402	1.84766
C	0.69288	1.11063	1.67901
N	1.56756	0.28666	1.00215
H	1.51508	-3.74854	-1.79775
C	2.67326	-2.02172	-0.86864
C	1.153	2.22755	2.49645
H	2.56772	0.34688	1.08123
C	3.75924	-1.04751	-0.54942
O	2.99637	-3.05694	-1.44841
O	2.50528	2.32146	2.49522
O	0.44878	2.98876	3.10383
C	4.85888	-1.31241	0.28813
C	3.92859	0.23822	-1.05003
H	2.72822	3.06309	3.06833
H	5.05664	-2.21909	0.83683
C	5.65294	-0.1821	0.28742

Continued on next page

Table S6 – continued from previous page

Atom Name	X	Y	Z
C	3.09372	0.99885	-1.98507
N	5.07987	0.74244	-0.53008
C	6.90951	0.05776	1.005
O	3.60082	2.22988	-2.19855
O	2.08893	0.59529	-2.50102
H	5.45486	1.65649	-0.73198
O	7.39961	1.286	0.73877
O	7.44164	-0.72822	1.737
H	3.01749	2.67186	-2.82562
H	8.2222	1.37703	1.23297

Table S7: Comparison of bond lengths calculated using DFT and MD simulations after energy minimization of DHICA-eumelanin.

Bond	DFT(Å)	MD after EM (Å)	Diff%
H1-O1	0.94	0.95	-1.06
O1-C1	1.36	1.35	0.48
C1-C2	1.49	1.47	1.32
C1-O2	1.22	1.20	1.38
C2-N1	1.38	1.39	-0.70
C2-C3	1.38	1.35	2.30
N1-H2	1.02	1.01	0.88
N1-C4	1.38	1.37	0.43
C3-H3	1.08	1.08	0.28
C3-C5	1.43	1.45	-1.61
C4-C5	1.40	1.47	-4.73
C4-C6	1.34	1.36	2.28
C5-C7	1.43	1.35	6.34
C6-C8	1.47	1.47	0.13
C6-C9	1.48	1.46	1.10
C7-C10	1.47	1.48	-0.59
C7-C11	1.52	1.49	1.93
C8-C12	1.41	1.38	2.40
C8-C13	1.46	1.42	2.93

Continued on next page

Table S7 – continued from previous page

Bond	MD after EM (Å)	DFT (Å)	Diff%
C9-C10	1.55	1.57	-1.21
C9-O3	1.24	1.21	2.27
C10-O4	1.23	1.20	1.76
C11-H4	1.09	1.10	-0.43
C11-H5	1.09	1.09	-0.02
C11-H6	1.08	1.09	-0.83
C12-O5	1.37	1.35	1.31
C12-C14	1.42	1.42	0.35
C13-C15	1.43	1.42	0.35
C13-C16	1.40	1.40	-0.07
O5-H7	0.95	0.96	-1.17
C14-O6	1.37	1.34	2.64
C14-C17	1.42	1.40	1.49
C15-H8	1.08	1.08	0.29
C15-C18	1.37	1.36	0.11
C16-C17	1.41	1.42	-0.70
C16-N2	1.37	1.37	0.21
O6-H9	0.96	0.99	-3.04
C17-C19	1.51	1.45	4.35
C18-N2	1.38	1.38	0
C18-C20	1.49	1.46	2.16
N2-H10	1.01	1.00	0.98
C19-C21	1.50	1.49	0.38
C19-O2	1.23	1.24	-0.62
C20-O8	1.35	1.36	-0.87
C20-O9	1.22	1.19	2.22
C21-C22	1.44	1.41	1.97
C21-C23	1.38	1.39	-1.09
O8-H11	0.95	0.96	-1.04
H12-C22	1.08	1.08	0.16
C22-C24	1.37	1.38	-0.79
C23-C25	1.5	1.472	1.90
C23-N3	1.37	1.36	0.84
C24-N3	1.37	1.36	1.06
C24-C26	1.49	1.47	1.62
C25-O10	1.36	1.35	0.90
C25-O11	1.22	1.19	2.81
N3-H13	1.01	1.01	0.56
C26-O12	1.36	1.35	0.53
C26-O13	1.23	1.20	1.76
O10-H14	0.95	0.96	-1.66
O12-H15	0.94	0.96	-1.78

Table S8: Comparison of angle degrees calculated using DFT and MD simulations after EM ensemble of DHICA-eumelanin.

**Angle**	**DFT(deg.)**	**MD after EM (deg.)**	**Diff%**
C1-N1-H1	125.79	124.99	0.63
C1-N1-C7	106.66	109.61	-2.69
C7-N1-H1	127.34	125.34	1.59
C2-C1-C9	124.00	126.51	-1.98
N1-C1-C9	124.59	122.15	1.98
N1-C1-C2	111.40	111.34	0.04
C1-C2-H2	124.05	125.20	-0.91
C8-C2-H2	128.18	127.84	0.26
C1-C2-C8	107.15	106.96	0.17
C8-C3-C26	120.53	125.40	-3.88
C4-C3-C8	114.50	117.06	-2.18
C4-C3-C26	121.07	117.54	3.00
C3-C4-C5	123.36	119.83	2.95
C3-C4-O1	125.6	121.47	3.40
C5-C4-O1	110.87	118.70	-6.59
C4-C5-C6	115.85	118.02	-1.83
C6-C5-O2	120.98	124.09	-2.50
C4-C5-O2	123.13	117.87	4.46
C7-C6-C12	124.12	122.38	1.42
C5-C6-C7	117.70	116.85	0.72
C5-C6-C12	118.17	120.75	-2.13
N1-C7-C8	109.25	106.17	2.90
N1-C7-C6	123.89	128.16	-3.33
C6-C7-C8	126.83	125.67	0.92
C2-C8-C7	104.42	105.91	-1.41
C2-C8-C3	135.49	131.62	2.93
C3-C8-C7	119.22	122.46	-2.64
O3-C9-O4	121.78	123.93	-1.73
C1-C9-O3	114.52	111.42	2.78
C1-C9-O4	123.00	124.65	-1.32
C9-O4-H3	106.87	107.16	-0.26
C10-N2-C16	109.33	109.33	0.00
C16-N2-H4	130.83	126.63	3.31
C10-N2-H4	119.37	123.83	-3.60
N2-C10-C11	109.45	109.20	0.22
C11-C10-C18	124.64	128.62	-3.09
N2-C10-C18	124.97	122.17	2.29
C17-C11-H5	125.18	127.91	-2.13
C10-C11-H5	127.59	125.37	1.77
C10-C11-C17	105.94	106.72	-0.73

Continued on next page

Table S8 – continued from previous page

Angle	MD after EM (deg.)	DFT (deg.)	Diff%
C6-C12-C13	121.52	120.83	0.57
C6-C12-C17	124.50	121.09	2.81
C13-C12-C17	113.98	118.08	-3.47
C12-C13-O5	121.52	120.66	0.71
C14-C13-O5	111.80	118.48	-5.63
C12-C13-C14	126.23	120.83	4.47
C13-C14-O6	117.00	114.23	2.42
C15-C14-O6	123.01	123.12	-0.08
C13-C14-C15	119.66	122.63	-2.41
C14-C15-C16	112.08	115.85	-3.25
C14-C15-C25	125.71	118.30	6.26
C16-C15-C25	121.97	125.81	-3.04
C15-C16-C17	128.68	121.70	5.73
N2-C16-C15	123.32	130.88	-5.78
N2-C16-C17	107.51	107.39	0.11
C11-C17-C12	132.96	131.75	0.92
C12-C17-C16	118.13	120.86	-2.26
C11-C17-C16	107.76	107.36	0.36
C13-O5-H7	106.78	107.96	-1.09
C14-O6-H8	115.16	107.29	7.33
C10-C18-O7	120.07	125.69	-4.47
O7-C18-O8	118.28	122.76	-3.65
C10-C18-O8	120.81	111.55	8.30
C18-O8-H6	109.25	106.48	2.59
C22-N3-H9	129.76	125.05	3.76
C19-N3-H9	124.06	125.42	-1.08
C19-N3-C22	106.15	109.53	-3.08
N3-C19-C23	123.22	122.93	0.23
N3-C19-C20	107.28	108.31	-0.95
C20-C19-C23	129.21	128.76	0.35
C19-C20-H9	109.11	107.15	1.83
C19-C20-H10	126.46	125.66	0.63
C21-C20-H10	122.41	127.19	-3.76
C20-C21-C22	106.11	107.05	-0.87
C22-C21-C25	131.68	127.95	2.91
C20-C21-C25	122.15	124.93	-2.22
N3-C22-C24	123.09	122.24	0.69
C21-C22-C24	125.63	129.80	-3.20
N3-C22-H9	111.11	107.96	2.92
C19-C23-O10	119.21	111.32	7.08
O9-C23-O10	120.32	123.80	-2.80
C19-C23-O9	120.45	124.88	-3.54

Continued on next page

Table S8 – continued from previous page

Angle	MD after EM (deg.)	DFT (deg.)	Diff%
C23-O10-H11	114.14	107.15	6.52
O11-C24-O12	119.09	123.65	-3.69
C22-C24-O11	119.32	125.22	-4.71
C22-C24-O12	121.52	111.13	9.34
C24-O12-H12	103.78	107.10	-3.10
C15-C25-C21	121.07	120.51	0.46
C21-C25-O13	122.70	117.31	4.59
C15-C25-O13	116.22	122.17	-4.87
H14-C26-H15	103.80	106.41	-2.45
H13-C26-H15	114.65	108.99	5.19
C3-C26-H15	106.35	110.21	-3.49
H13-C26-H14	106.56	108.92	-2.17
C3-C26-H14	111.50	110.14	1.23
C3-C26-H13	113.61	111.99	1.44
